# An Exploration of Gender and Age Differences in Psychosomatic Responses to Depression and Stress: An Analysis of Beck and DASS-42 Scales

**DOI:** 10.1192/j.eurpsy.2025.1337

**Published:** 2025-08-26

**Authors:** W. Wójtowicz, A. Zielazek, Z. Wydrych, N. Wyroba, N. Wojdacz, P. Omylak, K. Krysta

**Affiliations:** 1 Department of Rehabilitation Psychiatry, Medical University of Silesia, Katowice, Poland

## Abstract

**Introduction:**

This research investigates gender differences in psychosomatic responses among individuals of various ages, who had been diagnosed with depressive or anxiety disorders, or were in remission. It evaluates symptoms using the Beck Depression Inventory–Second Edition (BDI) and DASS-42 scales in a cohort of 30 adults in an outpatient clinic environment.

**Objectives:**

The aim of the study was to demonstrate the relationship between the degree of development of nicotine and the severity of symptoms related to emotions and mood with patients suffering from schizophrenia.

**Methods:**

A total of 30 adult participants (ages 21-69) were assessed using the BDI and DASS-42 scales. The study is focused on somatic symptoms (as measured by specific DASS-42 items) and their relationship to gender, age, and the severity of depression. The severity of depression was classified into mild, moderate, and severe categories based on BDI scores.

**Results:**

The study revealed that depression significantly affects both daily functioning and emotional responses to stress, with individuals suffering from severe depression showing psychosomatic symptoms most often, regardless of gender. The findings revealed that women diagnosed with depression reported higher rates of psychosomatic symptoms, such as dry mouth, breathing difficulties, and increased heart palpitations, compared to men. Men, on the other hand, exhibited difficulties in emotional regulation in response to stress, which can potentially indicate a general sense of insecurity and anxiety. Younger female individuals, under the age of 40, diagnosed with depression according to the BDI, exhibited more intense psychosomatic symptoms compared to older female patients. Furthermore, within the same cohort of women under 40, the intensity of psychosomatic symptoms was significantly higher in comparison to men in the same age group.

**Conclusions:**

The study uncovered the strong and interconnected relationship between stress and depression. Moreover, it indicated that the severity of psychosomatic symptoms associated with depression and stress is influenced by gender and age. As a result, it is crucial to adopt a comprehensive and personalized approach when treating patients, considering the severity of their conditions. The BDI and DASS-42 scales are effective in capturing these differences, highlighting their usefulness in both clinical and research contexts.

**Disclosure of Interest:**

None Declared

